# A Tale of Two Atria

**Published:** 2010-03-05

**Authors:** Khalil Kanjwal, Asma Khaliq, Blair P Grubb, Warren Foster, Yousuf Kanjwal

**Affiliations:** Division of Cardiology, Section of Electrophysiology, The University of Toledo Medical Center

**Keywords:** bi-atrial orthotopic cardiac transplant, intracardiac electrogram

## Abstract

We present an interesting intracardiac electrogram of a dissimilar atrial rhythm in a patient of bi-atrial orthotopic cardiac transplant.

## Case Description

**Figure 1:**
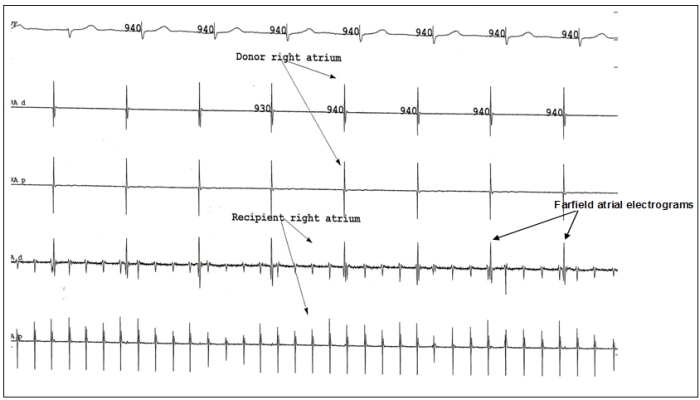
Intacradiac recording showing atrial flutter in recipient atrium and sinus rhythm in donor atrium. Far field potentials from donor atrium are recorded in recipient atrium.

Seventy eight year old man with prior history of bi-atrial orthotopic cardiac transplant for end stage ischemic cardiomyopathy presented with symptomatic typical counter-clockwise atrial flutter. He underwent successful ablation of cavo-tricuspid isthmus in the donor atria followed by dual chamber pacemaker implantation for a prolonged HV interval > 100 msecs. While attempting to implant right atrial pacing lead a dissimilar rhythm was recorded from the donor and the recipient atria. While the donor right atrium was in the sinus rhythm at the rate of 64 beats per minute (940 milliseconds), the recipient atrium was in a regular tachycardia at a rate of 300 beats (200 milliseconds) per minute suggestive of atrial flutter (Figure1). There was complete electrical isolation noted between the donor and recipient atria. The atrial lead was finally placed in the donor atrium, which revealed a sinus rhythm.

The donor atrium in patients with bi-atrial cardiac transplant tends to lie in a more septal and posterior position requiring steerable catheters to assure proper lead positioning. While the two atria tend to be electrically isolated, breakthrough conduction of atrial arrythmias of recipient atria across the suture line into the donor atrium has been reported [1]. Bidirectional atrio-atrial conduction with shift of sinus rhythm from the donor to the recipient atrium and back has also been reported [2].

It has been noted that in many bi-atrial transplanted hearts the recipient atrium is often in some form of supraventricular tachycardia. Sinus rhythm in the recipient atrium and atrial flutter in the donor atrium has also been reported [3,4].

The case highlights the importance of knowing the type of cardiac transplant procedure the patient has undergone to facilitate the proper catheter and pacemaker lead placements in these patients.
